# Complete mitochondrial genome of the deep pugnose ponyfish *Secutor ruconius* (Perciformes: Leiognathidae) in the East China Sea

**DOI:** 10.1080/23802359.2019.1670108

**Published:** 2019-10-15

**Authors:** Yanming Sui, Bo Qin, Xuefeng Song, Wenquan Sheng, Bianbian Zhang

**Affiliations:** Key Laboratory of East China Sea Fishery Resources Exploitation, Ministry of Agriculture; East China Sea Fisheries Research Institute, Chinese Academy of Fishery Sciences, Shanghai, China

**Keywords:** *Secutor ruconius*, mitochondrial genome, phylogenetic analysis

## Abstract

The complete mitochondrial genome sequence of *Secutor ruconius* is firstly described in this article. The total length of mitogenome is16,465 bp. It contains 13 protein-coding genes, 22 tRNA genes, and 2 ribosomal RNA genes. The overall base composition of H-strand is 31.58% A, 28.65% C, 25.23% T, and 14.53% G, with an A + T bias of 56.81%. The phylogenetic analysis result showed that the *S. ruconius*, *Zebrasoma flavescens*, *and Pristipomoides multidens* were closely related.

The deep pugnose ponyfish *Secutor ruconius* belongs to genus *Secutor* in family Leiognathidae of Chaetodontiformes. As a schooling demersal omnivore, *S. ruconius* plays an important role in reflecting any effects of harbor building activities. *S. ruconius* is one of the dominant benthic species in the North Bay of New Caledonia and Pagbilao of Philippines (Pinto 1988; Wantiez et al. 1996).

The complete mitochondrial genome of *S. ruconius* first determined in this paper was expected to provide help on population genetics of *S. ruconius* and further molecular phylogenetic studies.

The sample of *S. ruconius* in this article was collected from the East China Sea (123°56′E and 33°52′N). And the specimen is stored in the Key Laboratory of East China Sea Fishery Resources Exploitation, Ministry of Agriculture. According to genes from *S. ruconius*, such as 12S ribosomal RNA gene (Accession: KY352498), 16S ribosomal RNA gene (Accession: EU741807), cytochrome c oxidase subunit I (*COI*) gene (Accession: EF609613), primers were designed, and PCR amplification and sequencing were conducted.

The whole length of *S. ruconius* mitogenome was 16,465 bp and submitted in GenBank (Accession No. MN251862). The nucleotide composition of the heavy strand was 31.58% for A, 28.65% for C, 25.23%for T, and 14.53% for G, with a high A + T bias of 56.81%. It contains 13 protein-coding genes, 22 tRNAs and 2 rRNAs. Most genes were located on the heavy strand, but *ND6* and 8 tRNA genes (*tRNA^Gln^*, *RNA^Ala^*, *tRNA^Asn^*, *tRNA^Cys^*,*tRNA^Tyr^*, *tRNA^Ser^*, *tRNA^Glu^*, *tRNA^Pro^*) were encoded on the light strand. Most protein-coding genes initiated with ATG except for COI starting with GTG. It is also important to note that the majority of protein-coding genes (7 of 13 genes) is inferred to terminate with an incomplete stop codon T or TA– (*ND2*, *COII*, *ATPase 6*, *COIII*, *ND3*, *ND4*, *and Cyt b*); five protein-coding genes share the typical termination codon TAA (*ND1*, *COI*, *ATPase 8*, *ND4L*, *and ND5*); *ND6* uses TAG as a stop codon. The length of 12S (located between *tRNA^Phe^*and *tRNA^Val^*) and 16S (located between *tRNA^Val^* and *tRNA^Leu^*) rRNA genes were 950 bp and 1692 bp, respectively.

To investigate the phylogenetic relationship, we downloaded the mitochondrial genome sequences of 16 currently available Eupercaria. The concatenated sequences of 13 protein-coding genes, 2 rRNAs genes, and 22 tRNAs genes were aligned with the ClustalW program (Larkin et al. 2007). Using the maximum-likelihood (ML) method (Stamatakis 2006), the phylogenetic tree was constructed (Figure 1) by MEGA6 (Tamura et al. 2013). The best-fitting model (GTR + I + G) was obtained as the optimization model by jModelTest (Posada 2008). The result indicating that the *S. ruconius*, *Zebrasoma flavescens*, and *Pristipomoides multidens* were closely related (Figure 1).

**Figure 1.**
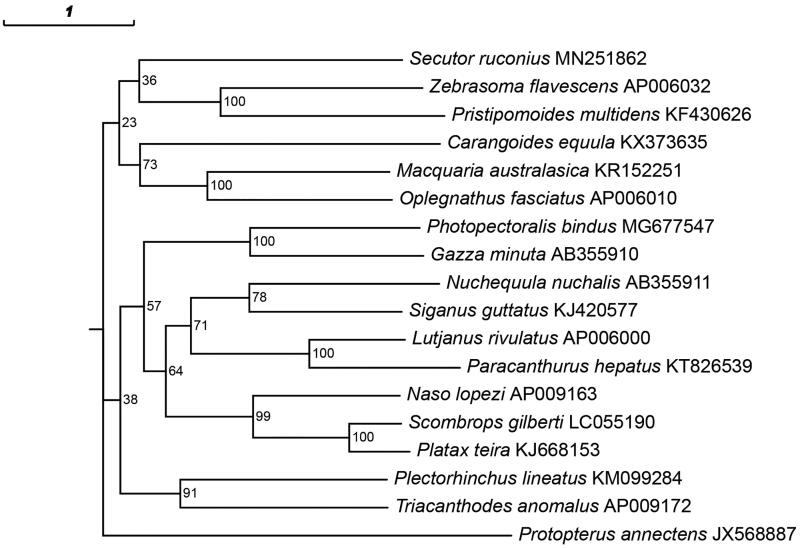
The phylogenetic tree based on the 13 protein-coding genes, 2 rRNAs genes, and 22 tRNAs genes of *Secutor ruconius*, *Zebrasoma flavescens*, *Pristipomoides multidens*, *Carangoides equula*, *Macquaria australasica*, *Oplegnathus fasciatus*, *Photopectoralis bindus*, *Gazza minuta*, *Nuchequula nuchalis*, *Siganus guttatus*, *Lutjanus rivulatus*, *Paracanthurus hepatus*, *Naso lopezi*, *Scombrops gilberti*, *Platax teira*, *Plectorhinchus lineatus*, *Triacanthodes anomalus and an outgroup Protopterus annectens*. The bootstrap (1000 replicates test) supports for maximum-likelihood (ML) method is indicated at each branch.
